# Association between personal values in adolescence and impaired bonding relationship with children

**DOI:** 10.1186/s40359-020-00463-9

**Published:** 2020-09-11

**Authors:** Erika Obikane, Kazuhiro Watanabe, Daisuke Nishi, Norito Kawakami

**Affiliations:** Department of Mental Health, Graduate School of Medicine, The University of Tokyo, The University of Tokyo, 7-3-1 Hongo, Bunkyo-ku, Tokyo, 113-0033 Japan

**Keywords:** Bonding disorder, Parent-child relationship, Personal value, Commitment to value

## Abstract

**Background:**

Bonding disorders happen as parents cannot build an affective relationship with children and are associated with their psychological and social conditions. Personal values impact well-being and psychological outcomes; however, how personal values influence bonding relationships is unknown. The purpose of the study was to investigate the relationship between personal values in adolescence recalled by adult participants and impaired bonding using a community-representative database.

**Methods:**

We conducted a retrospective study using cross-sectional data of adults living with children in Japan. Personal values were evaluated by value priorities measured by 11 items from Personal Value Questionnaires, and commitment to values measured by a Japanese version of the Personal Values Questionnaires II. Impaired bonding was evaluated by five items from a Japanese-version of the Mother-to-Infant Bonding Scale. Odds ratios of value priorities and commitment to values for impaired bonding relationships were calculated after adjusting covariates.

**Results:**

Of 13,920 people selected by probability proportionate sampling, 466 participants with children under 6 years old were selected for analysis. Personal values on improving society, graduating from school, positive evaluation from others, and pursuing one’s interest were negatively associated with impaired bonding relationship, while personal values on financial success were positively associated with impaired bonding relationship. Commitment to values were negatively associated with bonding problems.

**Conclusions:**

While a future longitudinal study is needed, the present findings may indicate that personal values in adolescence are associated with their bonding relationship with children.

## Background

Bonding disorders occur as parents cannot construct an emotional tie with their children. Past studies described disturbances in the parents’ relationship with their children, including the absence of affection, hate, rejection, neglect, or often impulses to harm [1, 2]. Attachment theory by Bowlby described a conceptual framework that the parent-child attachment relationship builds the foundation for empathy, sympathy, and prosocial attitudes and behaviors, and its influence lasts throughout life [3, 4].

Previous studies demonstrated that impaired parental bonding was associated with an increased risk for abusive parenting [5] and emotional and social problems of children [6, 7]. Children with secure attachment, on the other hand, presented fewer internalizing and externalizing problems [8, 9], developed better emotion regulation strategies [10], and made more satisfactory interpersonal relationships [11] . Moreover, impaired bonding with parents has long-term consequences. Some studies reported that impaired parental bonding was associated with psychiatric symptoms such as depression and anxiety in adulthood [12–16].

Past literature discussed that mothers who failed to bond to their child shared certain characteristics. Psychological and social factors of mothers play an important role for bonding disorders, such as depression [17–19], maternal anxiety [20], or poor social support [21], as well as perinatal conditions of mothers such as primiparity [18], unintended pregnancy [22, 23], and unplanned Cesarean section [24]. Paternal risk factors included paternal depression [25–28], paternal intimate partner violence against the mother [26], mother-to-child impaired bonding [26], maternal psychological distress [26], and presence of the father at birth [29, 30]. Pediatric risk factors included preterm birth [31] and sleep problems of infants.

In recent years, there is a growing interest for why people engage in certain behaviors. People act according to their reasons or motivations that are directed toward their personal values. Personal values are defined as broad, trans-situational, and desirable goals that serve as guiding in people’s lives [32]. Past studies reported that personal values are developed during adolescence through the psychological process in which adolescents learn to control the conflict between learned values and actual behaviors through self-regulation [33]. Personal values have been analyzed in two perspectives: the contents of values and the commitment to values. Past studies showed that the contents of values have been associated with well-being and health outcomes such as depression and suicidal behaviors [34–36]. Commitment to values has been positively associated with well-being, and negatively associated with suicidality [36, 37]. As personal values are associated with well-being and psychological symptoms, and bonding relationships are influenced by well-being and psychological factors of parents, personal values developed during adolescence may influence bonding with children. Moreover, recent studies suggested the link between attachment styles and prosocial values. Specifically, individuals with secure attachment reported higher scores on self-transcendence and self-directions. Based on Bowlby’s attachment theory [3, 4], they discussed that individuals with secure attachment are more likely to respond to needs of others, show more compassion and empathy for others, thus leading to prosocial values [38–40]. While emerging evidence suggests that attachment is associated with prosocial values, it is possible that values attained during adolescence may affect bonding relationships with the next generation. To our knowledge, no study has examined the association between personal values of adolescents and impaired bonding relationships with children.

Previous research on child-parent relationship described that parents in Japan developed unique relationship with their children from those in Western countries [41–43]. According to the study comparing parenting styles of Japan and the United States, Japanese parenting style emphasized accommodation while the Western parenting style emphasized individuation [43]. During infancy, Japanese parents tend to meet needs of their babies before they are expressed, characterized as “symbiosis of mother and child”. In the United States, infants are treated as separated individuals, and parents provide secure base and encourage the child to explore the outside the world [44–46]. In childhood, Japanese parents emphasize the importance of empathy, obligations, and fulfill expectations from others, while the U.S. parents emphasize the importance of expressing oneself and acquiring skills to negotiate with others [46]. As Japanese parenting style tends to place importance to social control and accommodation with others, individuals with prosocial values and attitudes may tolerate stereotype parenting styles in Japan and may present better bonding relationship.

This study aims to investigate the association between personal values during adolescence based on retrospective recall and impaired bonding relationships of parents with their children using an existing database of households in Japan. The study serves as a preliminary study to know if there was any relationship between personal values during adolescence and affective relationship with the offspring, which may stimulate a future longitudinal study on this topic.

## Methods

### Study design, setting, and participants

A retrospective study was conducted using cross-sectional data (wave 1, wave 2, and wave 3) from the Japanese Study on Stratification, Health, Income, and Neighborhood (J-SHINE), a panel survey conducted to examine the relationship between social determinants and health outcomes in urban and suburban municipalities in and around Tokyo from 2010 to 2017 [47]. The original study included adults aged 25 to 50 years, who had a child less than 18 years old at registry living in four municipalities (two sites in Tokyo metropolitan areas and two sites in neighboring prefectures). The current study included adults who had a child less than 6 years old at the time of child survey (2012). Participants were chosen by probability proportional sampling. The study excluded adults on the registry who could not be contacted due to death, address not identified, or absence for a long period. This study also excluded if any exposure, outcome, or confounding variables were missing. The J-SHINE study primarily had a target population of 549,249 people, and 13,920 people were selected for this study according to probability proportionate sampling. The wave 1 survey reached 8408 adults and presented valid data for 4357 adults (valid response rate, 31.3%). Among participants who completed the wave 1 survey, 2244 households had children under 18 years. In the wave 2 survey, 1520 households completed a child survey (valid response rate, 67.7%). Wave 3 in 2017 was completed by 4294 participants (valid response rate, 60.5%). After completing a written informed consent, participants were asked to answer questionnaires via a computer-aided personal instrument (CAPI). This study extracted demographic and psychosocial factors of adult participants from the wave 1 participants (2010–2011), personal value and commitment questionnaire from the wave 3 participants (2017), and bonding questionnaires from the wave 2 participants (2012), and used the data as cross-sectional data for analysis. The study protocol was approved by the Research Ethics Committee of the Graduate School of Medicine and the Faculty of Medicine, The University of Tokyo, Japan (No.630–73,361). All participants had given informed written consent to participate in the study.

### Measures

#### Personal values

Personal values in adolescence were evaluated by using value priorities and degrees of commitment to the values. For value priorities, we used the following 11 items (Table 1) that were suitable for measuring personal value priorities in adolescence from the 57-item Portrait Values Questionnaire (PVQ-57) [48];: avoiding causing trouble; positive evaluation; belief; financial success; improving society; pursuing one’s interest; social influence; enduring active challenging; cherishing family and friends; graduating from school; and stable lifestyle. Participants were asked to recall their value priorities at age 15 by the question “When you were 15-16 years old, how important did you think the following values were in your life?” They were asked to rate each value priority on a 7-point Likert scale (1 = *Not at all*, 7 = *Very important*).

**Table. 1:**
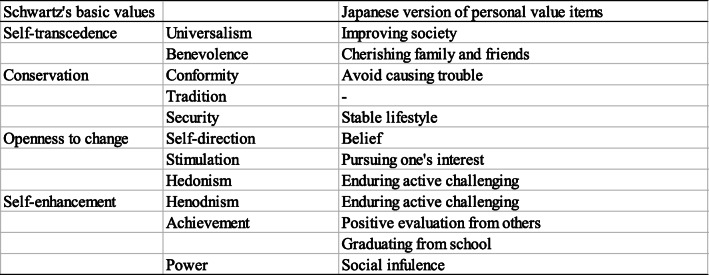
Schwartz’s basic values and personal value items used in the study

Commitment to the values was measured using a Japanese version of the Personal Values Questionnaires II (PVQ-II [49];, which was modified from the original version [50] and consisted of eight items. The PVQ-II has been confirmed of good validity and reliability (Cronbach alpha of the total score 0.71) [49]. Just as with value priority questionnaires, participants were asked to recall their commitment at age 15 and answer the following question “How committed are you to living this value?” The items were rated on a 5-point Likert scale to calculate the sum of the scores, with a higher score indicating greater commitment to the value.

#### Bonding disorders

Impaired bonding relationship was measured by a Japanese version of Mother-to-Infant Bonding Scale (MIBS-J), which is known to have reasonable reliability and validity for both mothers and fathers [51, 52]. Of 10 original items, five items that had been evaluated by confirmatory factor analysis [53] and commonly used by public health care settings were used for the study. The following items were included: “Feel loving towards my child (reversed item)”; “Feel scared or panicky when I have to do something for my child”; “Feel nothing toward my child”; “Enjoy doing things for my child (reversed item)”; and “Wish I did not have my child.” Participants were asked to choose an answer that described their feeling from: 0 = *Very much or most of the time*, 1 = *Very much so, some of the time*, 2 = *Slightly, some of the time*, and 3 = *Very rarely or not at all*. Following a previous study [53], participants with scores 0 to 1 were categorized as feeling maternal attachment, and those with scores 2 to 3 were categorized as having insecure maternal attachment.

#### Covariates

Sex, age, and education attainment were measured in wave 1 and used as covariates. Age was categorized into 25–30 years; 31–40 years; and 41–50 years. Education attainment was dichotomized as any or no college or university education.

### Post hoc analysis

As no previous study examined a relationship between personal values and bonding disorders, we would conduct a post hoc analysis to estimate the statistical power (1-β) by using G*Power version 3.1.9.2. In a post hoc analysis of a two-sided test with an odds ratio of 1-point increase of personal value scores for impaired bonding relationship as 0.82, H0 (the probability of the event [impaired bonding relationship]) as 0.24, sample size as 466, R2 = 0.1 and α = 0.05, the exact power (1-β) was 0.48.

### Statistical analysis

We conducted a multivariate logistic regression analysis to evaluate the association between personal values in adolescence recalled by adult respondents and impaired bonding relationship toward their offspring. Personal value priorities and commitment to values were entered as continuous values, and bonding scales were entered as binary values. Odds ratios of personal value priorities for insecure bonding attachment were calculated and adjusted for sex, age, and education attainment. Odds ratios of commitment to personal values for bonding disorders were also calculated and adjusted for covariates. All statistical analyses were conducted using SAS version 9.4 (SAS Institute, Cary, North Carolina, USA).

## Results

Of 13,920 people selected according to probability proportionate sampling, data of 466 adult participants with children less than 6 years old who had valid data for both the child survey and the wave 3 survey to report personal values variables were subject to analysis.

Table 2 presents demographic characteristics of the participants, mean scores of personal value priorities and commitment to values, as well as percentage of participants whose responses suggested the possibility of bonding disorders. According to Table 2, 5.8% of participants indicated insecure maternal attachment for the item “Feel scared or panicky when I have to do something for my child,” and 1.5% of participants were assessed as having insecure attachment for the item “Feel nothing toward my child.”

**Table 2:**
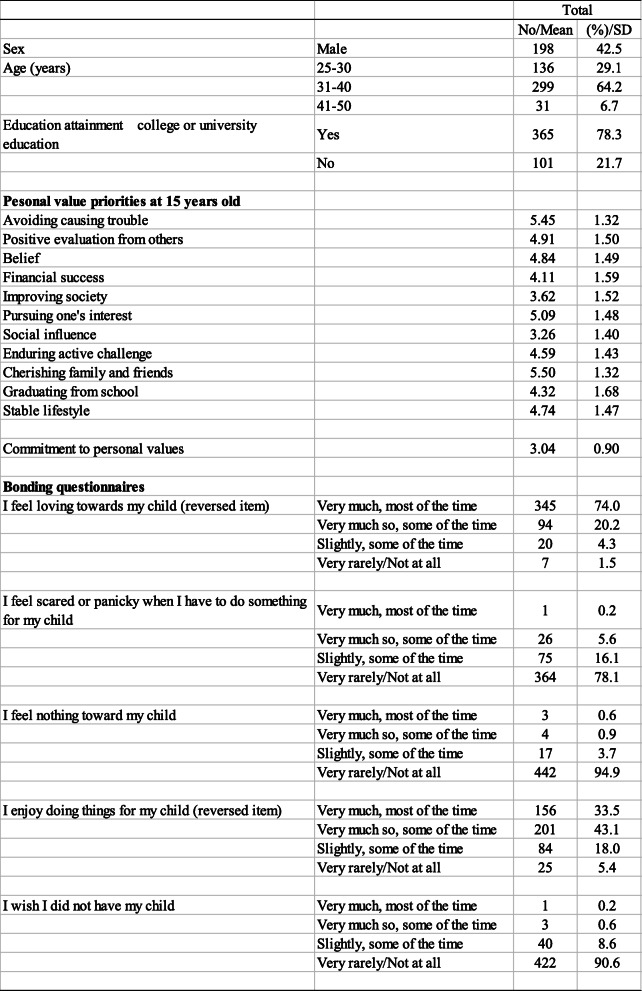
Demographic characteristics of participants

Table 3 indicates odds ratios of personal values at age 15 (value priorities and commitment to values) for bonding disorders with their offspring after adjusting covariates.

**Table 3:**
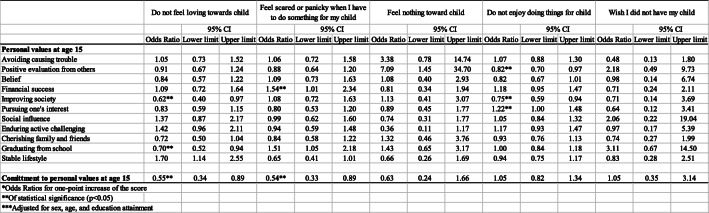
Odds ratios of personal values at age 15 and commitment for insecure bonding relationship with their children*

*Odds Ratios for one-point increase of the score

**Of statistical significance (*p* < 0.05)

***Adjusted for sex, age, and education attainment

For value priorities, values on improving society and graduating from school were negatively associated with not feeling loving toward child. Values on financial success showed positive association with feeling scared or panicked when participants had to do something for their child. Values on positive evaluation from others and improving society were significantly associated with enjoying doing things for one’s child, while pursuing one’s interest was negatively associated with not enjoying doing things for one’s child. Moreover, commitment to personal values at age 15 showed negative association with not feeling love toward one’s child and feeling scared or panicky when asked to something for child. In contrast, commitment to values showed negative association with feeling scared or panicky when asked to do something for child and feeling nothing toward child.

## Discussion

The study found that value priorities (improving society, graduating from school, and positive evaluation from others) and commitment to personal values in adolescence assessed by retrospective recall were significantly and negatively associated with parents’ impaired bonding relationship with their children; value priority on financial success was positively associated with impaired bonding relationship. A careful interpretation of these findings is required because the personal values and commitment to values during adolescence were measured based on retrospective recall by adult participants, and the measurement may be biased and could also be influenced by present personal values of the participants. However, the present study first provided preliminary evidence of a possible relationship between personal values during adolescence and bonding impairment with the offspring in the general population. A future longitudinal study should be promising to investigate the relationship between parents’ personal values in adolescence and their bonding relationship with their offspring.

According to the past literature, Sagiv and Schwartz categorized personal values as “healthy” and “unhealthy” values [35]. They argued that growth values (self-direction, benevolence, universalism, achievement, and stimulation) would enhance well-being, while deficiency values (conformity, tradition, security, and power) would undermine well-being, because pursuing these goals indicated people felt unsafe or threatened in their relations. Many studies reported positive association between growth values and subjective well-being [34, 35, 54]. Other literature has suggested that values focused on intrinsic goals (goals aimed for personal growth, emotional intimacy, or community involvement) are positively associated with well-being. Contrarily, values focused on extrinsic goals (goals aimed for financial success, social image, and fame) have been negatively associated with well-being [55]. Given that parental well-being influenced bonding relationship with their children, our finding was consistent with the past literature, as growth values motivated for intrinsic goals such as stimulation, achievement, and universalism were negatively associated with bonding disorders. Moreover, because growth values emphasize tolerance and welfare of other people and society, parents who affirmed growth values in adolescence may look dedicated and acceptable parents after having children and thus receive more support and emotional resources that contribute to their bonding relationship. Our study also demonstrated that financial success, which could be classified as a deficiency value (power) motivated for extrinsic goals, was positively associated with bonding disorders. Past studies discussed that people giving priority to deficiency values such as conformity, security, and power values were those who were feeling unsafe and preoccupied with lack of control, and this gives a rise to negative sense of well-being [35, 56].

Many emerging studies showed the link between these two [38–40]. The attachment theory stated that parent-child attachment relationship sets the foundation for empathy, sympathy, and prosocial attitudes and behaviors, and continues to have impact throughout the lifespan [3, 4]. Supporting our hypothesis that personal values would be associated with bonding relationships with the offspring, the present findings showed that prosocial value such as improving society was negatively associated with impaired bonding relationship with the offspring. The present study may also provide a piece of evidence on the association between attachment relationship and prosocial values.

The finding of the study should be examined in a cultural context as well. Past literature showed that Japanese parents presented uniqueness in their parenting style as compared to Western cultures [41–46]. Japanese parenting style emphasized the importance of empathy, obligations, meeting expectations of others, as opposed to Western parenting style emphasized expressing oneself effectively [43, 46]. As Japanese parenting style tends to place importance on accommodation with others or society, parents with prosocial values may accept parenting strategies more easily than those with less importance on prosocial values. Further studies examining the association between values and bonding impairment in regard to cultural context are expected.

Although our study showed significant association with some of the value priorities and impaired bonding relationship, our study did not show significant association between many of values priorities specially on cherishing family / friends and impaired bonding relationship, in contrary to our prediction. One possible explanation is that bonding relationship is influenced by numerous factors, and the effect of personal value on cherishing families and friends was not too large.

For commitment to personal values, several studies have demonstrated a relationship between commitment to values and well-being [37, 57]. One study reported a negative association between commitment to values and suicidal ideation [36] . Assuming that an affective relationship with offspring is influenced by well-being and psychological symptoms of parents, our finding fits with the previous literature that commitment to personal values in adolescence was negatively associated with bonding disorders in adulthood.

Our study, however had several limitations. First, the study used cross-sectional data; thus, it was subject to causation bias. Second, participants were asked to answer a questionnaire based on their recollection of personal values held at age 15; thus, it was subject to recall bias. Bonding relationship with the offspring at survey may affect the recall of personal values in adolescence. If the recall of personal values in adolescence was affected by present personal values, it is possible that present personal values are a more important determinant of bonding relationship with the offspring. A longitudinal study is needed to exclude these possibilities. Third, childhood adversity or severe mental illness from childhood may have been confounding factors as they influence both personal values in adolescence and bonding disorders in adulthood. Forth, the study used five of ten items from the original MIBS-J to assess bonding relationship with a child. Although the use of five items from the MIBS-J is widely used in previous research and public health care setting, we considered that these results should be interpreted with caution. Following the previous recent study, we investigated each item and used the same method for evaluating insecure attachment [53]. Finally, bonding relationship may be influenced by various factors, and we could not measure all of them; thus, it was subject to confounding bias. Future studies are expected to explore how personal values influence bonding disorders using longitudinal data.

## Conclusion

The study demonstrated personal values (stimulation, universalism, and achievement) and higher commitment to values during adolescence recalled by adult respondents were negatively associated with impaired bonding relationship in adulthood. Understanding how personal values in adolescence influence parents’ affective relationship with their offspring may contribute to the development of further strategies for preventing bonding disorders.

## Data Availability

The data that support the findings of this study are available from the corresponding author on request.

## Abbreviations

J-SHINE: Japanese Study on Stratification, Health, Income, and Neighborhood
CAPI: Computer-aided personal instrument
PVQ-57: 56-item Portrait Values Questionnaire
PVQ-II: Personal Values Questionnaires II
MIBS-J: Mother-to-Infant Bonding Scale
SD: Standardized deviation
CI: Confidence interval
